# Premature Moustache As Presenting Symptom of Nonclassic Congenital Adrenal Hyperplasia due to 2 Uncommon Mutations of the *CYP21A2* Gene

**DOI:** 10.1155/2011/913020

**Published:** 2011-07-06

**Authors:** Guy Massa, Philippe Gillis, Marianne Schwartz

**Affiliations:** ^1^Department of Paediatrics, Jessa Ziekenhuis, B 3500 Hasselt, Belgium; ^2^Department of Clinical Genetics, Rigshospitalet, University of Copenhagen, 2100 Copenhagen, Denmark

## Abstract

A Turkish boy was referred at the age of 3 6/12 years for the evaluation of a premature moustache. No other signs of virilisation were present. The endocrine evaluation led to the diagnosis of nonclassic congenital adrenal hyperplasia. Genetic analysis revealed 2 rare mutations of the *CYP21A2* gene, the gene encoding for the 21-hydroxylase enzyme: a recently reported R132C mutation in exon 3 and a R339H mutation in exon 8, both reported in the nonclassic CAH. An early moustache, for which the term premature moustache can be coined, can be the presenting symptom of nonclassic CAH. In all children presenting with a sex or age inappropriate development of a moustache, an endocrine evaluation is indicated.

## 1. Introduction

During normal male puberty, facial hair growth begins at a mean (SD) age of 14.9 (1.5) years [1, 2]. The first manifestation is the appearance of dark pigmented hair at the corner of the upper lip [2], for which the term “moustache” can be coined. Facial hair growth beginning before the age of 11 years (<*‒*2 SDS) has to be considered as “premature or precocious moustache” and is a symptom of virilization due to age inappropriate androgen levels, which can be from adrenal, gonadal, or exogenous origin [3]. 

Inherited adrenal enzymatic deficiencies causing hyperandrogenic symptoms some time after birth are defined as nonclassic congenital adrenal hyperplasia (NCAH), in contrast to the classic or congenital form of the disease in which clinical features are apparent at birth [4, 5]. Defects in 21-hydroxylase account for more than 90% of patients with NCAH. The clinical features, resulting from androgen excess, consist of premature pubic hair, hirsutism, acne, growth acceleration, advanced bone age, clitoromegaly in females, and penile growth in males. We here report the case of a 3-yr-old boy in whom a premature moustache led to the discovery of a NCAH. 

## 2. Case Report

The patient is the son of nonconsanguineous parents of Turkish origin. Family history revealed only familial hypercholesterolemia in the mother's family. After an uneventful pregnancy, the child was born at term with a birth weight of 2,315 g and a birth length of 47 cm. At birth, a partial cleft left lip and a complete cleft right lip and palate were found. The patient had several surgical corrections during the first 18 months of life. 

**Figure 1:**
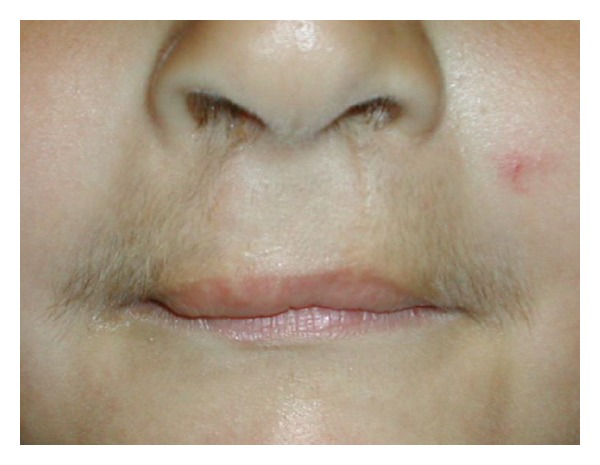
Upper lip of the patient at age 3 6/12 yrs. Note the moustache with dark pigmented hair over the upper lip and the scars of the cleft lip operations.

At the age of 3 6/12 years the patient was referred because of the presence of a moustache. Height was 101.7 cm (+0.5 SDS) and weight 15.5 kg (−0.1 SDS). After an initial catch-up growth between birth and the age of 2 years, height followed the +0.5 SD-line, in agreement with target height of 183.2 cm (+0.4 SDS). Bone age, according to Greulich and Pyle [6] was 4.0 yrs. Pubertal score according to Tanner [7] was A1P1G1, with only some downy pubic hair, a testicular volume of 2 mL and penile length of 4.7 cm (−0.9 SDS [8]). There was no sebaceous skin. Blood pressure was 110/60 mmHg. Except the moustache with dark pigmented hair all over the upper lip (Figure 1) and the scars of the cleft lip operations, physical examination was normal. 

### 2.1. Biochemical and Hormonal Data

Hematological parameters and serum electrolytes were within normal limits. Early morning levels of adrenocorticotropine (ACTH: 26.8 pmol/L [<22 pmol/L]; normal levels between [  ]), adrenal androgens (dehydroepiandrosterone 7.6 mmol/L [<1.2 mmol/L]; androstenedione 1.26 nmol/L [<0.9 nmol/L]); as well as of 17-*α*-hydroxyprogesterone (72.7 nmol/L [<1.0 nmol/L]) were elevated. Serum concentrations of cortisol (491 nmol/L) and testosterone (<0.15 nmol/L) as well as plasma renin concentration (31.2 ng/L) were normal. After a 0.25 mg intravenous bolus of tetracosactide (Synacthen), cortisol levels increased slightly to 596 nmol/L, whereas 17-*α*-hydroxyprogesterone levels increased to 140.8 nmol/L [<6.0 nmol/L]. Baseline and stimulated 17-*α*-hydroxyprogesterone levels were compatible with NCAH according to the nomogram of New et al. [9]. 

### 2.2. Genetic Analysis

**Figure 2:**
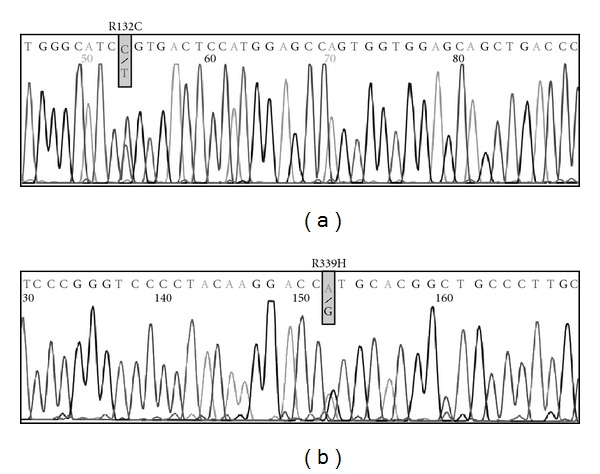
Results of *CYP21A2* sequencing of DNA of the patient. (a) panel shows part of the exon 3 sequence with the R132C mutation indicated. (b) panel shows part of the exon 8 sequence with the R339H mutation indicated. Both mutations are present in a heterozygous state.

**Figure 3:**
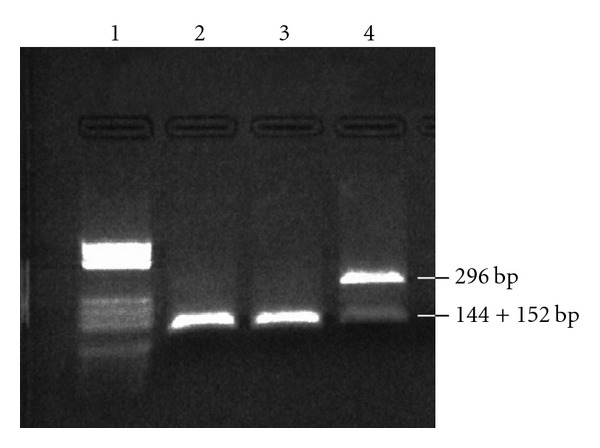
Results of restriction analysis. Lane 1: molecular weight markers; lane 2: normal control; lane 3: the mother of the patient: lane 4: the patient. The wild-type sequence has a *ApaI* site resulting in two fragments of 152 bp and 144 bp (are not separated on the gel). The mutation destroys this site, leaving the PCR product of 296 bp uncut.

Genomic DNA was extracted from peripheral lymphocytes of the patient and his mother after informed consent. The father was not examined. The *CYP21A2* gene, encoding for the 21-hydroxylase enzyme, was PCR amplified in two segments as described by Ohlsson and Schwartz [10] using Expand Long Template PCR System (Boehringer Mannnheim). PCR products were visualized on a 1% ethidium bromide stained agarose gel and purified with JET quick from Genomed. The two segments were sequenced in both directions, using an Applied Biosystems 310 automated DNA sequencer ABI Prism and BigDyeTM terminator Cycle sequencing Ready Reaction Kit from AB Applied Biosystems, according to the manufacturer's specifications. The obtained sequences were compared to the normal sequence, and in the patient 2 mutations were found: R132C in exon 3 and R339H in exon 8 (Figure 2). The observed mutations were confirmed by resequencing (R132C) or by restriction analysis (R339H). A PCR product of 296 bp was obtained by the following primers: R339H-F: 5′-TTGCTGAGGGAGCGGCTGGA, and R339H-R: 5′-AGCCTAGACACCCCTGGGTT, spanning the site of the R339H mutation. The mutation destroys a *ApaI* restriction site, while the normal sequence is cut into two fragments of 144 + 153 bp. The normal sequence has a *ApaI* site 144 + 152 bp. Restriction of the PCR product from the patient confirmed the heterozygosity, showing both 296 (mutant) + 144 + 152 (wild-type) (Figure 3). The mother was heterozygous for the R132C mutation, but did not carry the R339H mutation. 

## 3. Discussion

The presence of a moustache at the age of 3 6/12 years in our patient led to the diagnosis of nonclassic congenital adrenal hyperplasia, confirmed by biochemical and genetic analysis. Interestingly, no other clinical signs of androgen excess were present. Penile size, growth, and bone maturation were normal. Moreover, the patient did not have any disturbance in sodium balance, and during the surgical interventions for the cleft lip and palate no signs of adrenal insufficiency occurred, demonstrating that aldosterone and cortisol synthesis are not compromised to any clinically significant degree, as expected in most cases of NCAH [5].

Early morning levels of 17-*α*-hydroxyprogesterone and adrenal androgens were elevated and 17-*α*-hydroxyprogesterone levels further increased to levels seen in NCAH after administration of ACTH [9]. As basal levels of 17-*α*-hydroxyprogesterone can be normal in NCAH [11], an ACTH stimulation test remains an important tool in the evaluation of children with premature moustache or other signs of sex or age inappropriate virilization.

In our patient, 2 mutations of the *CYP21A2* gene were found. As in any autosomal homozygous disorder, 21-hydroxylase deficient NCAH arises only when mutations resulting in the production of an aberrantly functioning or absent 21-hydroxylase are present in both homologous *CYP21A2* genes. Although NCAH is considered a homozygous recessive disorder, the majority of the patients are compound heterozygotes, with a NCAH mutation on one *CYP21A2* gene and a severe mutation on the other allele [5]. Several mutations in *CYP21A2* resulting in NCAH have been described [4, 5]. The missense mutation, V281L, accounts for at least one of the *CYP21A2 *alleles in most of the NCAH patients. Other missense mutations associated with NCAH include P30L, P453S, and R339H [4, 5]. These mutations result in an approximately 50–80% decreased 21-hydroxylase activity. Functional studies have shown that the R339H mutation we found in our patient decreases enzymatic activity for 17-*α*-hydroxyprogesterone to 66% of normal [12]. 

The R132C mutation was only recently reported in an Argentinean woman with NCAH [13]. No second mutation was found in this patient; so this patient must have a heterozygote form of NCAH. The enzymatic activity of the mutated gene product has not yet been studied. As R132 is part of a cluster of basic amino acids involved in the redox partner interaction with P450 oxidoreductase, mutations in these basic amino acids are likely to cause electrostatic disturbances, and an impairment of the enzyme biological activity by the C132 mutation would be expected [14]. The magnitude of this impairment must be severe as it led to clinical symptoms and elevated 17-*α*-hydroxyprogesterone levels in the reported patient. As this mutation was not present in the mother of our patient, it can be inherited from the father or has arisen *de novo*. Unfortunately, the father could not be reached for genetic analysis.

In conclusion, the development of a premature moustache, for which the term premature moustache can be coined, can be the presenting symptom of NCAH. In all children presenting with a sex or age inappropriate development of a moustache, an endocrine evaluation is indicated.
